# Implementing Strength after Breast Cancer (SABC) in outpatient rehabilitation clinics: mapping clinician survey data onto key implementation outcomes

**DOI:** 10.1186/s43058-020-00060-2

**Published:** 2020-08-05

**Authors:** William A. Calo, Shawna E. Doerksen, Katherine Spanos, Mackenzi Pergolotti, Kathryn H. Schmitz

**Affiliations:** 1 Department of Public Health Sciences, Penn State College of Medicine, 500 University Drive, Mail Code CH69, Hershey, PA 17033 USA; 2 Penn State Cancer Institute, Hershey, PA USA; 3 Dartmouth College, Hanover, NH USA; 4 ReVital Cancer Rehabilitation, Select Medical, Mechanicsburg, PA USA; 5 Department of Occupational Therapy, Colorado State University, Fort Collins, CO USA

**Keywords:** Implementation outcomes, Online training, Physical therapy, Oncology rehabilitation, Breast cancer survivor, Exercise, Evidence-based guidelines

## Abstract

**Background:**

While 3.5 million breast cancer survivors in the USA are indicative of promising disease-free survival, many experience adverse effects in recovering from treatment. Evidence-based exercise programs may be a low-cost, easily disseminable solution to the challenge of recovering from adverse treatment affects. Therefore, after establishing efficacy in a large randomized controlled trial, we developed the Strength after Breast Cancer (SABC) program and the accompanying online course for clinicians interested in physical therapy to learn to deliver this rehabilitative exercise program to individuals with breast cancer. We surveyed clinicians who took the course to assess implementation of the program in outpatient rehabilitation clinics.

**Methods:**

Ninety-six clinicians completed the survey between June and December, 2017 (24% response). Guided by Proctor’s implementation outcomes framework, the respondents were asked if they had implemented (adoption) and are still implementing the program (sustainability), and which programmatic components they implemented (fidelity). Respondents were asked how many patients completed the program (reach), how patients got into the program (reach), the program’s delivery format (appropriateness), and whether clinics were reimbursed by third-party payers (cost). Finally, respondents were asked what barriers they faced in delivery of SABC (feasibility) and whether others in the clinic completed the course (penetration).

**Results:**

Seventy-six percent of respondents implemented SABC and among those, 93% (68/73) were still delivering it. All programmatic components were implemented by over two thirds of respondents (67–95%). On average, the program was delivered to 13 patients per clinic by the time respondents took the survey. Most patient referrals were from oncology clinics (50%). The majority of clinicians delivered SABC one-on-one (96%) and 72% of clinics were compensated via third-party payers. Major barriers were lack of referrals from oncologists (40%) and clinic’s competing demands (33%). We found no differences (Fisher’s exact test *p* > .05) in reported barriers between those who implemented the program and those who did not.

**Conclusion:**

Our findings suggest that the online training was sufficient to successfully implement the SABC program in outpatient rehabilitation clinics with high levels of adoption, fidelity, reach, and capacity for sustainability. Information on patient acceptability, cost-effectiveness, and how to overcome implementation barriers are still needed.

Contributions to the literature

- Evidence-based guidelines recommend regular exercise to recover after breast cancer treatment and ease adverse treatment effects but few US women have access to exercise rehabilitation programs in the communities they live.
- After establishing efficacy in a large randomized controlled trial with 295 breast cancer survivors, we developed the Strength after Breast Cancer (SABC) program, an online course to train clinicians interested in exercise rehabilitation to deliver SABC to their patients with breast cancer in outpatient rehabilitation clinics.
- Our findings suggest that the online training was sufficient to successfully implement the SABC program in outpatient rehabilitation clinics with high levels of adoption, fidelity, reach, and capacity for sustainability.

## Background

There are more than 3.5 million women in the USA who have undergone treatment for breast cancer [1]. Although increases in disease free survival are certainly good news, there are persistent adverse treatment effects that cause significant morbidity. Surgery often results in loss of upper body strength, which can vary depending on the extent of surgery and length of recovery [2, 3]. Surgery can also damage the lymphatic system, leading to lymphedema, which is characterized by swelling, changes in function, and increased risk for systemic infection [4]. Up to 30% of breast cancer survivors will develop lymphedema [4]. Cancer-related fatigue and weight gain are other common complaints among women undergoing chemotherapy for breast cancer [2, 5–7]. Radiation therapy has systemic effects similar to chemotherapy, particularly with regard to fatigue [5]. In addition, radiation causes permanent damage to the healthy soft tissue exposed to ionizing radiation, altering upper body function over time [8]. These adverse effects of treatment are widespread. In a previous study of 183 breast cancer survivors, we found that 62% had at least one, if not two, of the adverse treatment effects noted above 6 years post-diagnosis [2].

Evidence-based guidelines from the National Comprehensive Cancer Network [9], the American Cancer Society [10], and the American College of Sports Medicine [11] recommend regular exercise to recover after breast cancer treatment and ease adverse treatment effects. Our own work has contributed to the evidence base supporting the safety and efficacy of exercise programs after breast cancer treatment. The Physical Activity and Lymphedema (PAL) intervention, for example, assessed twice-weekly progressive strength training, including arm exercises, in breast cancer survivors 1 to 15 years post-diagnosis [12, 13]. In a large randomized controlled trial with 295 breast cancer survivors, PAL led to clinically meaningful improvements in upper body strength when compared to a no exercise control group [12, 13]. PAL also improved physical function, lymphedema symptoms, reduced likelihood of lymphedema onset or worsening, improved body image, appendicular skeletal muscle mass, lower body strength, and body composition [14, 15].

Based on these promising findings, our group adapted the PAL intervention into the Strength after Breast Cancer (SABC) program [16] and developed an accompanying online course which trains clinicians interested in physical therapy to deliver SABC to their patients with breast cancer in outpatient rehabilitation clinics. In 2015, we offered SABC nationwide through an online educational platform. As SABC was disseminated online and across the USA, we surveyed participants to assess whether they implemented the program after having taken the course. The present study evaluated the real-world implementation of SABC in outpatient rehabilitation clinics by mapping survey responses onto key implementation science constructs.

## Methods

### Participants

Participants were 96 clinicians who completed the SABC course online in 2015 and responded to our implementation assessment survey in 2017.

### SABC course

The online course was created by Dr. Kathryn Schmitz, in collaboration with Guenter Klose, and provided in 2015 through a popular online platform for physical therapy continuing education (Klose Training and Consulting website; http://klosetraining.com/course/online/strength-abc). Requirements to complete the course included being a licensed physical or occupational therapist, physician, or registered nurse. In addition, exercise professionals were offered the opportunity to have their credentials and experience with patients with cancer reviewed to discern eligibility. Upon registration and payment ($120), participants received a username and password; they had 3 months of access to the course. The 4-h course covered all aspects of setting up and running the SABC program including how to obtain referrals from oncology clinicians, screen potential patients, coordinate with a certified lymphatic therapist, educate patients about lymphedema, teach the 4-session exercise program, instruct patients on how to log their progress, motivate patients to perform exercises, handle logistical considerations, and manage discharge and wrap-up. The course also provided all the materials needed to set up the program in clinics, including PAL trial results, lymphedema education session in PowerPoint format, lymphedema risk-reduction guidelines, exercise instructions with photos, decision tree for tracking adherence, self-check list for program objectives, guidance for support staff, helpful information about billing codes, and weight training workout logs.

### Survey procedures

Klose Training and Consulting provided a list with the emails of those who completed the course (*n* = 395). Using REDCap, a secure web application for building and managing online surveys and databases, an initial email was sent to all individuals that included an invitation to complete the survey, information explaining the purpose of the survey, a link to access the survey online, and a statement about the confidentiality of their responses. Two weeks later, an email reminder was sent to those who had not yet responded the survey. The 10-min survey was conducted between June and December, 2017. No monetary incentive was offered to complete the survey. The survey response rate was 24%. The study was approved by the Institutional Review Board of the Penn State College of Medicine.

### Measures

Guided by Proctor’s Implementation Outcomes Framework [17], the survey assessed key indicators of implementation process and success: adoption, sustainability, fidelity, reach, appropriateness, cost, feasibility, and penetration. We chose this framework because it provides a systematic way to evaluate the implementation of innovations in healthcare settings and it is widely used in the literature to evaluate barriers and facilitators to intervention impact [18, 19]. The survey asked whether respondents implemented (adoption) and if they are still implementing (sustainability) the program in their clinics. Those who responded in the affirmative were asked which programmatic components they implemented (fidelity). Respondents chose components from the following list: evaluation by a certified lymphatic therapist, education about lymphedema, 4-session exercise program, symptom monitoring, patient’s motivation, resistance equipment for home exercise, and manage discharge. The survey also asked how many patients completed the program (reach) and how patients got into the program (reach). Response options were referrals from oncology clinics, clinic advertising, local media advertising, or others. The survey assessed whether the program was delivered one-on-one or a group format (appropriateness) and whether clinics were reimbursed by third-party payers (cost). Respondents also reported what barriers they faced to deliver SABC in clinics (feasibility); options were as follows: referrals, lack of patient interest, lack of interest from clinic management, third-party reimbursement, raising money to pay for therapist time, logistical difficulties, front desk staff training, competing demands, or others. The survey also asked whether others in the clinic completed the course (penetration). Respondents also reported what type of resistance equipment patients use at home for exercising (i.e., TheraBand resistance bands, dumbbells, household items, others) and whether the clinic provided the resistance equipment (feasibility).

### Data analysis

Descriptive statistics were used to describe participants’ characteristics and survey responses. We assessed whether actual implementation of SABC was associated with reported barriers. We also compared barriers identified by implementers with low versus high reach (determined by the mean number of patients per rehabilitation clinic who received the program as reported by survey participants). We used the Fisher’s exact test in the latter set of analyses exploring implementation barriers due to the small size of some cells. We analyzed survey data using Stata 14.0 (College Station, TX).

## Results

**Fig. 1:**
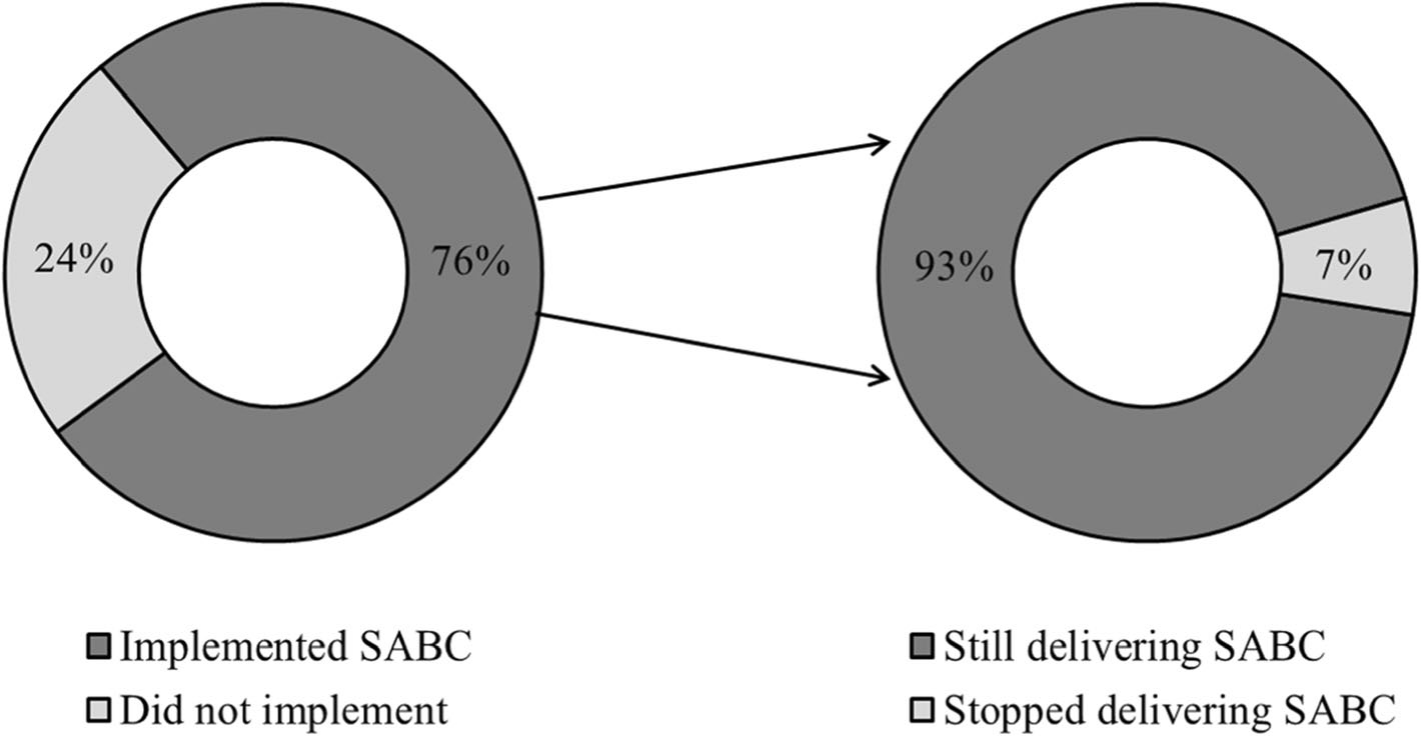
Implementation and sustainability of the SABC program in outpatient rehabilitation clinics

Seventy-six percent of respondents implemented SABC in their outpatient rehabilitation clinics and among those, 93% (68/73) were still delivering the program by the time they took the survey (Fig. 1). The majority reported implementing education about lymphedema (95%), discussing what resistance equipment to use for home exercise (92%), motivating patients (92%), and patient evaluation by a lymphatic therapist (90%; Table 1). All other programmatic components were implemented by over two thirds of respondents (67–86%). In those clinics that implemented SABC, on average, the program was delivered to 13 patients (range 1–60) per clinic. Patients found the program through referrals from oncology clinics (50%), ads posted in clinics (7%), local media advertising (2%), and other sources including referrals from other therapists and current participants (35%). The majority of respondents delivered the program one-on-one (96%) and a small number reported using a group format (11%). Almost three fourths of respondents (72%) were compensated for delivering SABC programming in their clinics via third-party payers.

**Table 1:** Programmatic components implemented by clinicians

Programmatic components	*N* (%)
Screen participants	49 (67)
Evaluation by a certified lymphatic therapist	66 (90)
Educate participants about lymphedema	69 (95)
Teach the exercise program in 4 sessions	55 (75)
Instruct patients on how to log their progress and monitor symptoms	63 (86)
Motivate participants	67 (92)
Help patient figure out what to use for resistance for home exercise	67 (92)
Manage discharge and wrap up	54 (74)

Respondents were able to check all that apply. Only those who reported delivering the SABC program (*n* = 73) got this question

The major barriers reported to delivering the program in clinics were lack of referrals from oncologists (40%), competing demands of physical and occupational therapists, administrators and front desk staff (33%), and logistical difficulties (29%; Table 2). We found no differences (*p* > .05) in reported barriers between those who implemented the program in clinics and those who did not. We also found no differences (*p* > .05) in barriers noted by implementers with low reach (< 13 patients) versus those reporting high reach (≥ 13 patients). The majority of respondents (83%) took the course as continuous education and more than one third (37%) reported that other therapists (range 1–10) in their clinics also completed the SABC online course. Respondents said patients with breast cancer used dumbbells of different weights (84%) for resistance exercise at home, followed by household items like soup cans (53%), resistance bands (29%), adjustable dumbbells (15%), and other equipment (7%). Only 15% of respondents said their clinics provided the resistance equipment to patients.

**Table 2:** Reported barriers to delivering the SABC program in outpatient clinics

Barriers	*N* (%)
Referrals from oncologist	38 (40)
Lack of interest from breast cancer survivors	16 (17)
Lack of interest from clinic management	13 (14)
Getting reimbursed by third-party payers	4 (4)
Raising money to pay for therapist time	4 (4)
Logistical difficulties	28 (29)
Front desk staff training	4 (4)
Competing demands of therapists, administrators, front desk staff	32 (33)
Others	15 (16)

Respondents were able to check all that apply. Results are combined for implementers and not implementers (*n* = 96) because there were no differences between those who implemented the program and those who did not (Fisher’s exact test *p* > .05)

## Discussion

With more women surviving a breast cancer diagnosis, there is a growing need for exercise rehabilitation programs to address the physical impact of adverse treatment effects. Despite the accumulated evidence showing that exercise improves fitness and strength after cancer treatment, women have limited access to supervised guideline-based exercise programs in the communities they live and work [11]. By mapping our evaluation data onto implementation science constructs, we found that the SABC program is a promising solution to increase the number of local cancer rehabilitation specialists delivering exercise therapy for oncology rehabilitation with high levels of adoption, fidelity, patient reach, and capacity for sustainability.

Over three fourths of respondents implemented SABC in outpatient rehabilitation clinics and among those, 93% were still delivering it. These high levels of adoption and capacity for sustainability may stem from staff values and a supportive organizational climate towards SABC. Klein and Knight suggest that successful implementation of new programs is a function of intervention-values fit [20]. The authors distinguish between unenthusiastic implementation, which occurs when a novel intervention fits poorly with staff values, and committed implementation, which occurs when an intervention fits well with staff values. With only 14% of respondents mentioning lack of interest from management as an implementation barrier, it is reasonable to assume that most rehabilitation clinics stimulated an organizational climate that made it appealing for clinicians to try implementing this new offering. The fact that over one third of survey participants reported that other clinicians took the SABC course also suggests that a positive organizational climate towards the program existed within clinics. In such a way, the encouraging climate for implementation would have reinforced the intervention-values fit for SABC. In addition, a high number of respondents said that third-party payers covered the costs for delivering SABC programming to patients. Cost reimbursement may have facilitated the initial adoption of the program, and subsequent sustainability, by relieving common concerns related to financial burden. Our prior work showed that insurance coverage for outpatient rehabilitation required careful review of the billing codes used and that copay amounts varied dramatically [16]. As funding and insurance coverage are major organizational barriers that limit exercise therapy becoming part of standard practice in oncology rehabilitation [21, 22], practitioners must work with insurers to set appropriate billing for these services.

Our evaluation also showed high levels of implementation fidelity with all core program components being implemented by over two thirds of respondents. Four out of the 8 components were implemented by 90% or more of respondents. Implementation fidelity is the degree to which an intervention is delivered as intended by the program developers [23] and is key to successful translation of evidence-based interventions into routine practice [24]. The SABC online course resulted from our own experiences implementing two large efficacy and effectiveness trials, respectively [13, 16]. These experiences guided us in developing a revised curriculum with the input of oncology care teams, physical therapists, and patients to improve the feasibility of implementation in real-life contexts. As such, revisions were intended to maintain high levels of implementation fidelity within community-based physical therapy settings without adversely affecting safety or effectiveness [25]. It is important to note that data were self-reported which poses limitations in terms of validity and accuracy and potential for self-desirability bias.

In those clinics that implemented SABC, an average of 13 patients per clinic received the program. This high level of reach suggests patient demand for exercise-based rehabilitation programs exists within outpatient settings. Such a demand may have incentivized therapists and clinics’ leaders to continue implementing SABC as suggested by the high sustainability rate reported in our study. Patient referrals were a very important contributor to this high reach. Half of the surveyed clinicians noted that patients who entered the SABC program did so because of a referral from an oncologist and another third said it was because of a referral from other therapists or existing patients. Interestingly, respondents also said that the most pressing barrier to delivering SABC in clinics was actually a lack of referrals from oncologists. Prior studies have noted that some clinicians have low awareness about the proven benefits of exercise as part of oncology rehabilitation [22]. Dennett et al. reported that some oncologists are still recommending rest for the management of fatigue [22]. This lack of awareness contributes to poor referral rates and has serious implications for future growth of exercise-based oncology rehabilitation. Patients may also need more education of how exercise can assist their rehabilitation needs. Further education to both oncology specialists and patients while building a trained workforce may lead to increased levels of reach.

Logistical difficulties and staff competing demands were also mentioned as important barriers to delivering SABC in clinics. One potential solution to these implementation challenges would be the use of a physical therapist champion to assist with administrative barriers. The original trial testing the effectiveness of the SABC program required this type of leader for successful implementation [16]. The implementation science literature defines a champion as someone who (a) is internal to an organization; (b) has an interest and commitment to implementing a change; (c) works persistently to drive implementation forward, even if no formal recognition or compensation was offered; (d) is enthusiastic, dynamic, energetic, personable, and persistent; and (e) has high levels of conviction [26]. In such a way, champions would take control of implementing the SABC program in their clinics to smooth over any manageable logistical issues. They would also support institutionalization of the program, including working with the billing office to secure feasible payment options, promoting the course to other therapists, and working with external oncology care teams to seek referrals.

In terms of strength, our study surveyed a geographically diverse number of clinicians who are at the front line of oncology rehabilitation delivery. Limitations include the use of self-reported measures to assess implementation outcomes; future studies should consider using observational methods (e.g., in-person assessment by external evaluator or recorded therapy sessions) for measuring implementation, particularly fidelity. Self-reported measures introduce the possibility of participation bias, where those therapists who implemented the SABC program were more likely to respond to the survey than those who did not. This could have contributed to the high adoption and fidelity rates reported by respondents. We also need additional data about two critical dimensions of fidelity: implementer adherence to program’s protocols and competence in delivering the program [24]. In addition, our study did not assess acceptability of the SABC program from the perspective of patients. More information is also required from oncologists to address why they do or do not refer patients to the program as well as their overall awareness of the importance of exercise therapy for improving oncology rehabilitation after treatment. The noted lack of referrals provides an opportunity to develop and test interventions to improve the care pathway of breast cancer patients and survivors from treatment to rehabilitation, especially in outpatient settings. Finally, although we collected some data on reimbursement by third-party payers, little is known about the cost-effectiveness of exercise-based programs in outpatient rehabilitation clinics, like SABC. Researchers should conduct cost-effectiveness analyses of the SABC program and especially evaluate the possible financial burden on patients since many of the clinics did not cover costs of exercise equipment for patients and prior studies show copay varies tremendously.

## Conclusion

Exercise is an effective way to manage common adverse effects of breast cancer treatment, and the SABC course proves a valuable tool to implement an evidence-based exercise program for patients to receive those benefits. Our findings suggest that the online training provided to clinicians interested in physical therapy successfully led to implementation of SABC in outpatient rehabilitation clinics, with high levels of reach, adoption, and fidelity. Because any new interventions will not be effective if they are not implemented well, the promise of SABC cannot be advanced without special attention to implementation science. Research is still needed to further understand implementation outcomes of this program, especially acceptability at the patient level and provider level, as well as what can be done to increase reach through engagement of oncology care teams.

## Data Availability

The dataset used and/or analyzed during the current study is available from the corresponding author on reasonable request.

## Abbreviations

SABC: Strength after Breast Cancer
PAL: Physical Activity and Lymphedema
